# Learning Something From Nothing: The Critical Importance of Rethinking Microbial Non-detects

**DOI:** 10.3389/fmicb.2018.02304

**Published:** 2018-10-05

**Authors:** Alex Ho Shing Chik, Philip J. Schmidt, Monica B. Emelko

**Affiliations:** ^1^Department of Civil and Environmental Engineering, Faculty of Engineering, University of Waterloo, Waterloo, ON, Canada; ^2^Institute of Hydraulic Engineering and Water Resources Management, Vienna University of Technology, Vienna, Austria; ^3^Department of Earth Sciences, Faculty of Geosciences, Utrecht University, Utrecht, Netherlands

**Keywords:** QMRA, microbial risk assessment, zeros, detection limit, censored data, presence-absence, pathogens

## Abstract

Accurate estimation of microbial concentrations is necessary to inform many important environmental science and public health decisions and regulations. Critically, widespread misconceptions about laboratory-reported microbial non-detects have led to their erroneous description and handling as “censored” values. This ultimately compromises their interpretation and undermines efforts to describe and model microbial concentrations accurately. Herein, these misconceptions are dispelled by (1) discussing the critical differences between discrete microbial observations and continuous data acquired using analytical chemistry methodologies and (2) demonstrating the bias introduced by statistical approaches tailored for chemistry data and misapplied to discrete microbial data. Notably, these approaches especially preclude the accurate representation of low concentrations and those estimated using microbial methods with low or variable analytical recovery, which can be expected to result in non-detects. Techniques that account for the probabilistic relationship between observed data and underlying microbial concentrations have been widely demonstrated, and their necessity for handling non-detects (in a way which is consistent with the handling of positive observations) is underscored herein. Habitual reporting of raw microbial observations and sample sizes is proposed to facilitate accurate estimation and analysis of microbial concentrations.

## 1. Introduction

Whether describing pathogens in water or the density of red blood cells, the concentration of discrete objects cannot be measured directly. In these cases, concentration is estimated by enumerating or detecting the objects in finite sample portions (e.g., volumes); such approaches are used extensively in health, food, and water applications. These estimates are required for decision making, during which they are typically evaluated against concentration-based criteria or targets (Dickey et al., 1999; Lund et al., 2000; Havelaar et al., 2001; Gerba and Rose, 2003; Gracias and McKillip, 2004; Koepke et al., 2007; Schijven and de Roda Husman, 2011; Davis, 2014; World Health Organization, 2017). This underscores the importance of accurate representation and analysis of detection- and enumeration-based data, especially where the protection of public health is at stake.

Regardless of application area, concentration estimates derived from non-detects (NDs) or low counts are widely perceived to be more uncertain and less reliable than those based on higher counts. This has often led to a desire to quantify enough of these objects by modifying the enumerated sample portion so that the count falls in a range that is deemed acceptable (Emelko et al., 2008; American Public Health Agency et al., 2017; United States Food and Drug Administration, 2017). When this is not possible, resulting NDs are widely reported as being less than a detection limit (e.g., <1 per analytical sample size) and used as a statement about true source concentration. This convention has been widely implemented and deemed precautionary because it usually leads to higher (i.e., conservative) mean concentration estimates. Approaches for handling this type of ND data are often developed out of computational convenience, though more elaborate approaches also continue to be developed. One important reason for the development of more complex approaches arises from the recognition that true microbe concentrations are imperfectly estimated by the analytical methodologies used to obtain counts from samples (Nieminski et al., 1995; Allen et al., 2000). For example, the impact of measurement error (e.g., random sampling error and imperfect and/or variable analytical recovery) on microbial concentration estimates has been widely demonstrated and thoroughly discussed (Nahrstedt and Gimbel, 1996; Gronewold et al., 2008; Gonzales-Barron and Butler, 2011; Schmidt and Emelko, 2011; Commeau et al., 2012; Pouillot et al., 2013; Duarte et al., 2015). Measurement error applies universally to all microbial detection and enumeration methods and refers to the random discrepancy between the actual concentration in the presumably homogeneous source and the concentration estimate obtained from a sample (Emelko et al., 2010). Failure to account for measurement error properly has been shown to bias concentration estimates and associated risk estimates, sometimes by orders of magnitude (Pouillot et al., 2013; Schmidt et al., 2013). In contrast, the implications of interpreting and handling NDs using approaches that mishandle measurement error have not been thoroughly discussed. Current reporting conventions for NDs frequently obfuscate their interpretation, so data analysis approaches have been tailored to how these data are reported rather than what the NDs truly represent.

Here, methods used to characterize microbial concentrations from detection- and enumeration-based data are reviewed, and common misconceptions associated with the reporting and handling of NDs are discussed. Examples that draw upon conventions and standards in the drinking water industry are provided to demonstrate why common approaches that treat NDs as censored data are incorrect and lead to bias in interpretation. Finally, recommendations to facilitate standardized reporting and analysis of such data are provided.

## 2. State of scientific practice

Microbial concentrations in food and water are often estimated using detection- and enumeration-based methods. A detection test produces either an ND or positive (≥1 microorganism) result. With a series of repeated presence/absence tests (e.g., Colilert Quanti-Tray®) and assumed Poisson-distributed numbers of microorganisms in each test (as a function of aliquot size and shared source concentration), the most probable number (MPN) approach yields a maximum likelihood estimate (MLE) of concentration (Pouillot et al., 2013). In these detection methods, reporting of raw aliquot sizes and presence/absence results is necessary for concentration estimation. Enumeration-based methods are distinguished from detection-based methods because they yield a whole number count of target microorganisms within an analytical sample size. These include cultivation plate counts of colonies or virus plaques and cell counts obtained using microscopy or flow-/solid-phase cytometry. We suggest that the concepts addressed in this paper also apply to increasingly common biochemical molecular methods (e.g., qPCR, 16s rRNA gene sequencing); however, such methods are excluded from the scope of this work due to additional assumptions and complexities in the inference of concentrations using these methods, which remain hotly debated (Keer and Birch, 2003).

Although many of the aforementioned microbial enumeration methods have been standardized, protocols for the representation, reporting, and analysis of resulting data remain largely inconsistent. Standard microbiological methods, such as those stipulated within *Standard Methods for the Examination of Water and Wastewater*, Part 9000 (American Public Health Agency et al., 2017), ASTM D5465-16 (American Society for Testing and Materials, 2016), ISO 8199:2005, and ISO 7218:2007/2013 (International Standards Organization, 2005, 2013), advise that observations should be reported as a count per analytical sample size (e.g., volume). These data (count and sample size) are *raw* in the sense that the original information pertaining to the precision of the count has not been lost, whereas neither the count nor sample size can be deduced when only a concentration estimate is reported (e.g., 1 microorganism in 64.4 L is more informative than just a reported concentration estimate of 0.0155 microorganisms/L).

In many cases, counts beyond certain thresholds are considered unreliable and avoided if possible. For example, when counting colonies in plating protocols (American Society for Testing and Materials, 2016; American Public Health Agency et al., 2017), an upper bound is often reasonably suggested because of overcrowding and difficulty in distinguishing between individual colonies. In these cases, an upper threshold is often applied beyond which a result of “too numerous to count” (TNTC) is reported. Notably, many conventions related to lower thresholds and NDs also exist. For example, some methods (e.g., in which counts are obtained from a dilution series) suggest that NDs should be omitted in concentration estimation (United States Pharmacopeial Convention, 2014). It has been a common convention to report NDs as <1 microorganism per analytical sample size (International Standards Organization, 2013; Forum on Environmental Measurements (FEM) Microbiology Action Team, 2016; United States Food and Drug Administration, 2017), which is the purported method detection limit (MDL). This is frequently interpreted at face value as the *de facto* concentration for statistical analyses and regulatory compliance—despite the recently stipulated caveat that MDLs are inapplicable to “methods that do not produce results with a continuous distribution such as […] presence/absence methods, and microbiological methods that involve counting colonies” (United States Environmental Protection Agency, 2016).

### 2.1. NDs in analytical chemistry

To understand the widespread convention of reporting NDs as values below MDLs in microbiology, it is important to understand the origin and motivation behind the concept of an MDL. The MDL (also known as the “limit of detection”) was developed as a performance criterion for chemical analyses (Glaser et al., 1981). This concept has remained largely unchanged since its original conception (Currie, 1999). Although slight variations of this concept exist, the MDL can be operationally defined as the minimum measurement of concentration of a substance that can be reported with a high degree of confidence (commonly 95 or 99%) that the concentration is actually greater than zero (Armbruster and Pry, 2008) (i.e., that the measurement is unlikely to be just random noise despite actual absence of the substance). In stark contrast to the field of microbiology where NDs reflect the inability to collect and/or observe a single microorganism in a particular analysis, analytical chemistry results are much less susceptible to influence by small numbers of analyte particles—signals obtained for quantification arise from the collective effect of very large numbers of atoms/molecules/ions per mole (e.g., 6.022 × 10^23^). In fact, merely 50 ng of lead in a liter of water (a detection limit attainable by current lead analysis methods) is comprised of more than 1.45 × 10^14^ lead atoms due to the magnitude of Avogadro's number. In chemistry, random sampling errors associated with specific numbers of analyte particles in a well-mixed sample is largely insignificant compared to errors introduced through the application of the analytical method itself—the accuracy of the measurement is limited by the precision of the measurement instrument. The construct of the MDL is intended to reflect these method-specific errors to facilitate comparisons of data generated using different analytical methods for the same analyte at the lower end of concentration ranges.

Although the MDL construct can be useful, concentration observations falling below these thresholds are not devoid of meaning and it has been recommended that these data should be reported as measured chemical detections. They are still valid observations from which true concentrations can be estimated (albeit with greater uncertainty) by applying appropriate statistical approaches (e.g., that make relevant assumptions concerning randomly distributed error, unbiased analytical methodology, and interference effects) (Analytical Methods Committee, 1987). However, some policies require substances to be described as “absent, present in only a limited number of samples, or present in less than a specified number or amount of a given quantity” (National Research Council (US NRC) Subcommittee on Microbiological Criteria, 1985) in regulatory and contractual frameworks, leading to the adoption of reporting limits (i.e., a value below which data are not reported) by many laboratories.

While these reporting conventions are not themselves problematic, they become problematic if these data are incorrectly interpreted or statistically analyzed. The implications of NDs in environmental chemistry have long been recognized (Analytical Methods Committee, 1987; Lambert et al., 1991). Unaltered zero concentrations preclude the calculation of geometric means and cannot be fit by many continuous distributions (without their explicit accommodation through a zero-inflated model). Values reported as below detection or reporting limits have commonly been either omitted or substituted with a function of the limit (Helsel, 2006) to facilitate computationally convenient analysis. These approaches are deemed conservative, but sacrifice information about data reliability and uncertainty that may be critical in decision making. Chemical concentration data reported as less than a detection limit are an example of censored continuous measurements (where the exact measured value within the specified interval is unknown), for which appropriate statistical approaches do exist (Helsel, 2005).

### 2.2. NDs in enumeration-based microbial methods

The direct application of analytical chemistry MDL concepts and associated censoring conventions to microbial enumeration data has inflicted similar challenges for statistical analysis in microbiology. Taking NDs as zeros and weighing them with other non-zero counts based on their respective analytical sample sizes is sufficient for the simple calculation of mean concentrations provided the microorganisms are randomly dispersed and a representative sample was obtained (i.e., from a source where the spatial distribution of the analyte is not heterogeneous); however, this approach is insufficient for fitting concentration distributions and quantifying data reliability or uncertainty in the calculated mean (Parkhurst and Stern, 1998). Commonly used omission and substitution methods borrowed from analytical chemistry for summarizing and reporting mean microbial concentrations in water introduce bias; substitution methods have been demonstrated to be increasingly biased with greater proportions of NDs in both chemical and microbial data (Parkhurst and Stern, 1998; Helsel, 2005; Roser and Ashbolt, 2005). While the bias introduced using substitution methods can offer a substantial safety factor when harmful microorganisms are rare (by considering them to be present when they are not or they have not been detected), it is critical to note that this bias offers no factor of safety when it is most needed (e.g., when pathogens are routinely observed) (Parkhurst and Stern, 1998).

The acknowledgement that “…[data reported as censored] cannot be treated statistically without modification” (American Public Health Agency et al., 2017) and the growing need to quantify uncertainty in the concentration estimate have led to the development of various statistical tools for analyzing these data. Critically, NDs in microbial data are in fact observed counts of zero commonly misrepresented as censored data. Their misrepresentation has led to the adoption of censored data approaches for handling microbial NDs (Lorimer and Kiermeier, 2007; Busschaert et al., 2010; Williams and Ebel, 2012). While many statistical analyses have assumed that microbial concentrations are measured directly and precisely, markedly different statistical methods have been developed that acknowledge the probabilistic relationship between actual observed data (including NDs) and the underlying microbial concentrations by accounting for measurement error (Nahrstedt and Gimbel, 1996; Gronewold et al., 2008; Gonzales-Barron and Butler, 2011; Schmidt and Emelko, 2011; Commeau et al., 2012; Pouillot et al., 2013; Duarte et al., 2015). As would be expected, different approaches for handling microbial NDs can result in substantially different outcomes. Specifically, the statistical analysis of inappropriately censored NDs may lead to erroneous microbial concentration estimates and subsequent interpretations—this is demonstrated by the examples below. It is critical to recognize that data for which *both* raw counts and sample sizes are *known* are not censored—these include NDs that are based on counts of zero in known sample sizes. These data are not censored and must not be statistically treated as such.

## 3. Results: evidence that microbial non-detects are not censored data

### 3.1. Occurrences of microbial NDs are not solely a function of analyte concentrations

All microbial concentration estimates are imprecise, not only NDs. An ND can arise when either the concentration is truly zero or when target microorganisms are present in the source but not successfully detected. Because of the latter case, it is commonly understood that an ND does not necessarily imply that the concentration is truly zero. Indeed, consistent with the aphorism “absence of evidence is not evidence of absence,” a concentration of zero cannot actually be proven by an ND for this reason.

**Figure 1:**
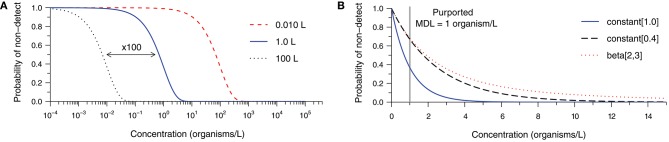
Probability of a non-detect observation as a function of organism concentration and **(A)** various analytical sample volumes given 100% analytical recovery, and **(B)** various analytical recovery profiles given a 1.0-L sample, each assuming Poisson random sampling error. The constant [0.4] and beta-distributed [beta(2,3)] recovery profiles share a mean of 40% analytical recovery, but the latter is more variable.

Figure 1 examines factors leading to ND results at non-zero concentrations (derivation in Supplementary Material S1). Figure 1A depicts the probability of observing an ND as a function of the true concentration and the sample volume assuming Poisson-distributed organism counts and a method with 100% analytical recovery. Probability of ND profiles are presented for volumes of 0.010, 1.0, and 100 L to illustrate the impact of 100-fold increases in the analytical sample size. Common sample volumes for total coliform/*Escherichia coli* and protozoan (oo)cyst analyses are 0.100 and 100 L, respectively. Intuitively, the probability of an ND observation from a single sample increases with decreasing concentration and analytical sample size. In practice, the occurrence of random NDs can be reduced by increasing sample size.

Building upon the previous example, Figure 1B addresses the occurrence of NDs given a 1.0 L sample volume and various analytical recovery profiles. The bold curve in Figure 1B is identical to the one in Figure 1A, but plotted on a linear concentration scale. It represents 100% analytical recovery, whereas the second curve addresses the scenario of a constant analytical recovery of 40% (i.e., the probability of observation for each microorganism initially gathered is 40% in any sample). Logically, the probability of NDs increases as microorganisms are more likely to be lost during sample processing. The remaining curve retains a mean recovery of 40%; however, substantial variation in recovery among samples is described by a beta distribution. This further inflates the probability of an ND observation because some samples would have relatively low recovery. Clearly, the occurrence of NDs is sensitive not only to the source concentration and the analytical sample size, but also the analytical recovery profile of the method for the particular sample matrix.

It may be useful to consider the concentration beyond which NDs become improbable (e.g., probability <1%) when comparing alternative methods, choosing a target sample volume, or determining the appropriateness of a method for a particular application. We propose that this threshold may be called a method sensitivity limit (MSL) because sensitivity is the probability of detection when the target microorganisms are actually present in the source. Considering the examples in Figure 1B and allowing for 1% probability of observing a non-detect, the scenario with 100% analytical recovery has an MSL of 4.6 organisms per liter. With 40% analytical recovery, the MSL increases to 11.5 organisms per liter. The MSL is 32.5 organisms per liter in the final scenario, illustrating the pronounced effect of variability in analytical recovery upon sensitivity of microbial analytical methods. While this calculated value could be useful, it is important to note that it is sensitive to uncertainty in the parameters and shape of the analytical recovery distribution (where low recovery values are common), and would not be practical to evaluate for every method and sample matrix.

### 3.2. Uncertainty in concentration estimates precludes MDL-based interpretation of results

**Figure 2:**
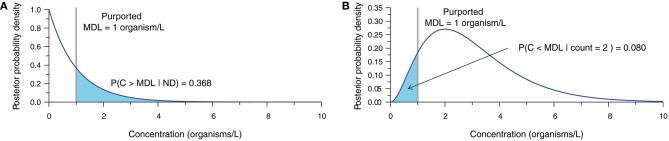
Posterior probability density function (PDF) characterizing uncertainty in the true concentration given **(A)** an ND observation, and **(B)** an observation of two organisms, each based on a 1.0-L sample, 100% analytical recovery, and a semi-infinite uniform prior. The purported MDL of 1 organism/L is shown with the probability of the true concentration exceeding or falling short of the purported MDL, respectively.

The statistical analysis of inappropriately censored microbial data ultimately leads to erroneous concentration estimates and subsequent interpretations. Bayesian techniques (Gelman et al., 2014) provide a means of demonstrating the uncertainty surrounding the concentration estimate obtained from microbial enumeration data (Gronewold et al., 2008; Gonzales-Barron and Butler, 2011; Schmidt and Emelko, 2011; Duarte et al., 2015). Accounting for measurement error, these methods describe the relative probability of alternative values of the true microbial concentration given the count observation obtained from the analytical sample and a prior representing beliefs about the plausible values of concentration before data analysis. Figure 2 illustrates what a single ND observation (Figure 2A) and an observation of two microorganisms (Figure 2B) within a 1.0 L sample volume imply about concentration assuming perfect analytical recovery and using a relatively uninformative semi-infinite uniform prior (derivation in Supplementary Material S2).

When an ND is observed (Figure 2A), there is still a large probability (≈ 37% in this example) that the actual concentration exceeds the purported MDL, therefore invalidating the assertion that an ND means that the actual concentration is <MDL. Conversely, a count of two organisms (Figure 2B) leads to a considerable probability (≈ 8% in this example) that the actual concentration could still be less than the purported MDL. This simple demonstration shows that the interpretation of NDs as censored data below the purported MDL is inappropriate, and further underscores that point estimates of concentration ought not be treated as exact measurements.

### 3.3. Censoring in detection- and enumeration-based microbial methods

Although NDs in microbial detection and enumeration methods are not censored data, there are scenarios in which certain microbial methods yield truly censored data. Censored data occur when there is incomplete knowledge about an observed measurement above, below, or between specified values (Millard et al., 2012). Such censoring can be inherent to the method or imposed deliberately by the analyst as exemplified below. In either case, censoring applies to raw measurements (e.g., count test results) rather than calculated values that are not measured directly (e.g., concentration estimates).

An ND in presence-absence tests (e.g., Colilert®, Rapid HiColiform™, AquaCHROM™) implies a count of zero within the associated analytical sample volume. A positive test result can be construed as an inherently censored count of at least one microorganism because the method cannot reveal the exact number of microorganisms leading to detection. For a series of presence-absence tests, MPN approaches implicitly reflect censored data analysis by using the cumulative probability of all non-zero counts (i.e., the complement of the probability of a ND) to represent a positive test result in the likelihood function.

When using culture-based methods, counts beyond an upper limit are conventionally reported as TNTC [e.g., 150 or 200 colony forming units (cfu) for spread plates, 80 cfu for membrane filtration, and 300 cfu for pour plates, American Society for Testing and Materials 2016; American Public Health Agency et al. 2017]. If a specific observed count is replaced with TNTC, then this is an example of imposed censoring. In contrast, censoring is inherent if counting is terminated upon reaching the limit or is not attempted because the count would clearly exceed it. Such truly censored observations may be incorporated into the Bayesian method described previously (or any likelihood-based method) using cumulative density for the censored range of counts rather than just probability density associated with particular observed counts. Some standards (American Public Health Agency et al., 2017) recommend completing a new analysis with dilution to *replace* TNTC results. We suggest that TNTC results should be *retained* in subsequent statistical analyses by using likelihood-based methods that allow inference from both the TNTC result and the count obtained through re-analysis of the sample. This would enable more accurate description of knowledge about the concentration by harnessing all of the available information rather than omitting inconvenient data.

## 4. Implications for policy and practice

Many environmental science and public health decision-making and regulatory frameworks rely upon the accurate evaluation of microbial concentrations and comparison with concentration-based criteria. For example, evaluation of source water pathogen concentrations is used to determine minimum treatment infrastructure requirements in the provision of safe drinking water (United States Environmental Protection Agency, 2006; Alberta Environment and Sustainable Resource Development, 2012). Here, concentration estimates that bias high may lead to misallocation of resources including costly infrastructure investments and operational adjustments.

*Giardia* has been the most commonly reported intestinal protozoan in North America and worldwide; it is also likely the most common cause of surface water-borne infectious disease outbreaks (Adam et al., 2016; Efstratiou et al., 2017). A set of eight source water *Giardia* cyst counts (Table 1) from the larger City of Calgary database were used to illustrate the potential impacts of various ND data analysis approaches. The impact of inappropriate ND data analysis approaches will vary in accordance to characteristics of each dataset; greater bias can be expected when NDs constitute a larger proportion of the dataset and perfect analytical recovery is not attainable. However, more detailed analysis regarding the scale of implications associated with such characteristics (e.g., number of samples, proportion of zeros, distribution of positive detections, etc.) was beyond the scope of the present investigation. Consistent with current practice and interpretation of the regulations, the raw data were not adjusted for viability or infectivity, with 100% analytical recovery assumed.

**Table 1:** Summary of raw water samples analyzed for *Giardia* cysts from City of Calgary, AB, Canada—October, 2012.

**Sample**	**1**	**2**	**3**	**4**	**5**	**6**	**7**	**8**
Raw count	1	0	0	0	0	0	0	2
Volume processed (L)	64.4	50.2	50.0	53.2	50.2	50.4	50.4	50.7
Data reported (cysts/100 L)	1.6	<2.0	<2.0	<1.9	<2.0	<2.0	<2.0	3.9

The data were used to obtain MLEs of the mean and standard deviation of *Giardia* cyst concentrations (United States Environmental Protection Agency, 2006; Alberta Environment and Sustainable Resource Development, 2012) assuming log-normally distributed concentrations and independence among sampling events. NDs were omitted in Approach A and substituted with the MDL and half the MDL in Approaches B and C (approaches critiqued by Helsel, 2005), and were handled as censored data in Approach D (Busschaert et al., 2010; Williams and Ebel, 2012). The purported MDL of one cyst per volume analyzed is critical for substitution and censored data methods. For Approach D, the cumulative density between zero and the purported MDL was used for NDs (Busschaert et al., 2010). Maximum likelihood estimation was applied for Approaches A–D using the *fitdistrplus* package (v. 1.0–9) (Delignette-Muller et al., 2017) in *R*. In Approach E, a Poisson distribution was used to account for random sampling error with log-normally distributed concentrations, using the *poilog* package (v. 0.4) (Grøtan and Engen, 2008) in *R* (details provided in Supplementary Material S3). It is important to note that Approaches A–D are based only on reported concentration estimates whereas Approach E (like other approaches that account for measurement error) necessitates the reporting of raw data. Statistics from this analysis are summarized in Table 2.

**Table 2:** Comparison of *Giardia* cyst concentration statistics obtained using various approaches for handling microbial NDs.

**Approach/Model**	**Treatment of NDs**	**$\hat{\mu }$**	**$\hat{\sigma }$**
(A) log-normal	Omitted	0.0276	0.0136
(B) log-normal	Substituted with MDL	0.0216	0.0055
(C) log-normal	Substituted with $\frac{1}{2}$ MDL	0.0139	0.0067
(D) log-normal	Censored data (<MDL)	0.0149	0.0100
(E) Poisson log-normal	Zeros with random sampling error	0.0071	0.0071

*$\hat{\mu }$: MLE of the mean cyst concentration (cysts/L)*.

*$\hat{\sigma }$: MLE of the standard deviation (cysts/L)*.

As would be expected, Approaches A–D yielded substantially higher mean *Giardia* cyst concentrations relative to Approach E because the NDs were omitted or represented as non-zero values. Omission and substitution approaches are known to lead to biased mean concentration estimates relative to methods appropriate for censored data (Helsel, 2005). However, the types of microbial ND data considered herein are fundamentally not censored, as discussed above. There is a critical difference between censored data approaches (Approach D) and those that actually incorporate NDs as legitimate, discrete observations by accounting for measurement error (e.g., Approach E). In this example, the parasite concentrations were overestimated relative to Approach E by a factor of 2.1–3.9 when NDs were inappropriately handled (Approaches A–D).

Given sufficient and suitable information, the MLE approach incorporating random sampling error (Approach E) can be extended to account for analytical recovery. However, model fitting by MLE becomes more difficult with increasing model complexity—numerical integration required for evaluating the resulting likelihood function becomes practically intractable in many cases. Bayesian methods can be used to fit more complex probabilistic models to data, but also suffer from substantial parametric uncertainty where insufficient data and/or data that are relatively uninformative about model parameters are available (Gleit, 1985; Helsel and Cohn, 1988). Indeed, small statistical sample sizes are often inevitable when using time- and/or resource-intensive microbial analytical methods [such as those for protozoan (oo)cyst enumeration (United States Environmental Protection Agency 2005, 2012)]. For example, utilities undertaking minimum source water monitoring requirements for the determination of drinking water treatment targets (Alberta Environment and Sustainable Resource Development, 2012) would be determining running mean *Giardia* cyst concentrations based on monthly samples collected over the course of 2 years (i.e., *n* = 24). The impact of small statistical sample sizes on concentration distribution parameter estimates is exacerbated when all of the data available are NDs, in which case statistical analysis is not possible without strongly subjective priors.

Although treatment requirements are not typically determined based on mean *Giardia* cyst concentrations estimated from a handful of samples, these data may exemplify monitoring results from utilities that draw upon high quality source waters. Such systems, especially those that have limited treatment, operational, and/or monitoring capacity, are particularly vulnerable to the implications associated with overestimated mean concentrations. As demonstrated in this analysis, concentration estimates may be biased high by a factor of two or more just by handling NDs as censored. This could lead to operational and maintenance costs/adjustments (e.g., energy for UV disinfection, alteration of design flow rates) (Cotton et al., 2001) that are inordinate given the levels of pathogens actually present in the source. Thus, such bias can also inappropriately affect assessments of water treatment plant “firm capacity,” which indicates pathogen treatment capacity in absence of one key treatment barrier and therefore informs infrastructure needs. While application of these approaches may result in bias that invokes more conservative levels of treatment (Parkhurst and Stern, 1998), it is better to analyze microbial concentrations accurately and apply consistent safety factors—regardless of the data—than to apply flawed data analysis approaches with unspecified safety factors attributable to preventable bias. This precludes the universal and equitable application of microbial standards, and ultimately undermines the consistent level of public health protection that the industry strives to maintain.

## 5. Conclusions

- Non-detect microbial detection and enumeration data are fundamentally not censored data and should not be reported or analyzed as such.
- Method detection limits are not intended to be used for, and have therefore been misapplied in, detection- and enumeration-based methods that count discrete microorganisms.
- The convention of reporting non-detects as censored values relative to a method detection limit is misleading when using enumeration-based methods and has resulted in the misuse of censored data statistical approaches for microbial data analysis.
- It is inconsistent to consider the uncertainty in non-detects by representing them as censored data while ignoring the inherent uncertainty in all non-zero counts.
- Censored data approaches should be reserved for data correctly interpreted as being censored, such as too numerous to count plate counts where the actual count is known only to exceed a specified threshold.
- This work re-emphasizes that raw microbial data must be reported to facilitate proper statistical analysis approaches that account for measurement error.

## Author contributions

PS, ME, and AC designed the work. AC, PS, and ME analyzed the data and prepared the manuscript.

### Conflict of interest statement

The authors declare that the research was conducted in the absence of any commercial or financial relationships that could be construed as a potential conflict of interest.
