# Supplementary material for: Learning Something From Nothing: The Critical Importance of Rethinking Microbial Non-detects
Source: Front Microbiol. 2018 Oct 5;9:2304. doi: 10.3389/fmicb.2018.02304 (PMC6182096; doi:10.3389/fmicb.2018.02304)
Supplement: Supplementary file 1 [file Data_Sheet_1.pdf]

Supplementary Material: Learning something from nothing: The critical importance of rethinking microbial non-detects

S1 CALCULATION OF THE PROBABILITY OF A NON-DETECT OBSERVATION

In this work, the number of microorganisms present within a sample volume V collected from a presumably homogeneous source with a true concentration of c can be represented as the Poisson random variable X:

$$P(X = x) = \frac{e^{-cV}(cV)^x}{x!} \tag{S1}$$

The probability of a non-detect observation assuming all microorganisms that are present are successfully recovered and enumerated is simply:

$$P(X = 0) = \frac{e^{-cV}(cV)^0}{0!} = e^{-cV} \tag{S2}$$

The product of the concentration and the volume provides the number of microorganisms that can be expected to be present. Therefore, the expected number of microorganisms for a recovery of r would be multiplied by this factor, as reflected in the exponent:

$$P(X = 0|r) = e^{-cVr} \tag{S3}$$

Recovery can also be a random variable R represented by the standard beta distribution with parameters a, b and the following characteristics:

$$E[R] = \frac{a}{a+b} \tag{S4}$$

$$Var[R] = \frac{ab}{(a+b)^2(a+b+1)} \tag{S5}$$

$$f_R(r) = \frac{\Gamma(a+b)}{\Gamma(a)\Gamma(b)} r^{a-1}(1-r)^{b-1} \tag{S6}$$

The probability of a non-detect considering the recovery profile as defined by the beta distribution is obtained by integrating over the range of possible recovery values, thereby obtaining the confluent hypergeometric function ${}_1F_1(a, b, c, V)$ ($hyperg1F1$ in R):

$$P(X = 0; a, b) = \frac{\Gamma(a+b)}{\Gamma(a)\Gamma(b)} \int_0^1 e^{-cVr} r^{a-1}(1-r)^{b-1} dr \tag{S7}$$

S2 EVALUATION OF THE POSTERIOR PROBABILITY DENSITY FUNCTION OF THE MICROBIAL CONCENTRATION BASED ON A SINGLE OBSERVATION

In this work, Bayesian techniques were used to evaluate the uncertainty in the true concentration. A relatively uninformative semi-infinite uniform prior ($f(c) = 1, c > 0$) on the concentration was updated with the additional information provided by the count observation (incorporated through its likelihood function) to obtain a posterior distribution. Based on Bayes theorem:

$$posterior \propto prior \times likelihood \tag{S8}$$

The likelihood function of the concentration is given by:

$$\mathcal{L}(c) = \frac{e^{-cV}(cV)^x}{x!} \tag{S9}$$

Therefore the posterior becomes:

$$P(c|x) \propto \frac{e^{-cV}(cV)^x}{x!} \tag{S10}$$

$$\propto (cV)^x e^{-cV} \tag{S11}$$

This posterior for concentration has the form of a gamma distribution with shape parameter $\rho = x + 1$ and scale parameter $\lambda = 1/V$.

S3 FITTING A POISSON LOG-NORMAL DISTRIBUTION TO *GIARDIA* CYST RAW DATA ASSUMING 100% ANALYTICAL RECOVERY

In this work, maximum likelihood estimates (MLEs) of arithmetic mean and standard deviation of the log-normal distribution within the Poisson log-normal model for count data were evaluated based on raw *Giardia* data (including both counts and sample volumes) and assuming 100% analytical recovery of the method (i.e., perfect sensitivity). Analytical recovery data were available for this dataset based on ColorSeed^{TM} internal seeding standards; however, such data are not widely available in practice for all drinking water treatment systems and therefore have not been widely mandated in the current interpretation and application of water regulations such as those specified in Alberta (Alberta Environment and Sustainable Resource Development, 2012). Variable analytical recovery and other factors influencing *Giardia* concentration estimates can be accounted for within hierarchical Bayesian frameworks, but is not considered here for simplicity and has been discussed elsewhere (e.g., Schmidt et al., 2013). The concentration c can be estimated if n samples with volumes $(V_i, i = 1, 2, ..., n)$ are assumed to be independent and collectively representative of the log-normally distributed concentration. If the cysts in the source are randomly dispersed and the sample is well mixed, then the number of cysts observed in each sample (X_i) is Poisson-distributed with mean cV_i, assuming all present cysts are successfully enumerated. By accounting for random sampling error, non-detects are integrated seamlessly with non-zero counts (and their respective analytical volumes).

$$ln(c) \sim N(\mu, \sigma^2) \tag{S12}$$

$$X_i \sim Poisson(\lambda_i) \tag{S13}$$

$$\text{where} \quad \lambda_i = cV_i$$

$$\text{and} \quad ln(\lambda_i) = ln(c) + ln(V_i)$$

By mathematical expectation,

$$E[ln(c)] = \mu \tag{S14}$$

$$Var[ln(c)] = \sigma^2 \tag{S15}$$

The expectation of the natural logarithm of λ yields:

$$\begin{aligned} E[ln(\lambda_i)] &= E[ln(c)] + E[ln(V_i)] \\ &= \mu + ln(V_i) \end{aligned} \tag{S16}$$

$$\begin{aligned} Var[ln(\lambda_i)] &= Var[ln(c)] + Var[ln(V_i)] \\ &= \sigma^2 + 0 = \sigma^2 \end{aligned} \tag{S17}$$

Therefore, the probability of the number of cysts observed in each sample provided the assumptions inherent in Equations S12 and S13 is Poisson log-normally distributed with mean $\mu + ln(V_i)$ and standard deviation σ. The probability density function can be solved numerically using the *poilog* package (v. 0.4)(Grøtan and Engen, 2008) in *R*, which was originally developed for the analysis of species abundance data in ecology. The *dpoilog* function within the package does not explicitly allow for the specification of "sampling intensity" (or in this case, the sample volume) as the sampling intensity is seldom known for species abundance data in many ecological applications. However, given mathematical expectation

properties (Equations S16 and S17), we can re-express the probability of the number of cysts observed in each sample in terms of μ, σ and the measured volume V_i:

$$P(X_i|\mu;\sigma;V_i) = dpoilog(X_i, \mu + ln(V_i), \sigma) \tag{S18}$$

Clearly, the unbiased estimates of μ and σ accounting for measured (i.e., known) sample volumes can be easily determined (V. Grøtan, *personal communication*, January 9th, 2018). The joint probability of all X_i observations gives the likelihood function that can be expressed as the product of the probability of each observation.

$$\mathcal{L} = \prod_{i=1}^{n} dpoilog(X_i, \mu + ln(V_i), \sigma) \tag{S19}$$

The resulting likelihood function can be maximized by testing values of the MLE parameters μ and σ iteratively.

REFERENCES

Alberta Environment and Sustainable Resource Development (2012). *Standards and Guidelines for Municipal Waterworks, Wastewater and Storm Drainage Systems.* (Edmonton: Alberta Queen's Printer)

Grøtan, V. and Engen, S. (2008). Poilog: Poisson lognormal and bivariate Poisson lognormal distribution. *R package version 0.4*

Schmidt, P. J., Emelko, M. B., and Thompson, M. E. (2013). Analytical recovery of protozoan enumeration methods: Have drinking water QMRA models corrected or created bias? *Water Res.* 47, 2399–2408
